# Genome skimming and exploration of DNA barcodes for Taiwan endemic cypresses

**DOI:** 10.1038/s41598-020-77492-2

**Published:** 2020-11-26

**Authors:** Chung-Shien Wu, Edi Sudianto, Yu-Mei Hung, Bo-Cyun Wang, Chiun-Jr Huang, Chi-Tsong Chen, Shu-Miaw Chaw

**Affiliations:** 1 Biodiversity Research Center, Academia Sinica, Taipei, 11529 Taiwan; 2 Department of Forensic Science Investigation Bureau, Ministry of Justice, New Taipei City, 231209 Taiwan; 3 School of Forestry and Resource Conservation, National Taiwan University, Taipei, 10617 Taiwan

**Keywords:** Genetic markers, Genome, Plant molecular biology, Conservation biology

## Abstract

Cypresses are characterized by their longevity and valuable timber. In Taiwan, two endemic cypress species, *Chamaecyparis formosensis* and *C. obtusa* var. *formosana*, are threatened by prevalent illegal logging. A DNA barcode system is urgently needed for reforestation and conservation of these two cypresses. In this study, both plastomes and 35S rDNAs from 16, 10, and 6 individuals of *C. formosensis*, *C. obtusa* var. *formosana*, and *C. obtusa* var. *obtusa* were sequenced, respectively. We show that the loss of plastid *trnT-GGU* readily distinguishes *C. formosensis* from its congeneric species. We demonstrate that entire sequences of plastomes or 35S rDNAs are capable of correctly identifying cypress species and varieties, suggesting that they are effective super-barcodes. We also discover three short hypervariable loci (i.e., 3′ETS, ITS1, and *trnH-psbA*) that are promising barcodes for identifying cypress species and varieties. Moreover, nine species-specific indels of > 100 bp were detected in the cypress plastomes. These indels, together with the three aforementioned short barcodes, constitute an alternative and powerful barcode system crucial for identifying specimens that are fragmentary or contain degraded/poor DNA. Our sequenced data and barcode systems not only enrich the genetic reference for cypresses, but also contribute to future reforestation, conservation, and forensic investigations.

## Introduction

*Chamaecyparis*, commonly named cypress or false cypress, is a small genus belonging to the Cupressaceae family in cupressophytes (also called conifers II). The cypress genus includes five to six species native to eastern Asia (Japan and Taiwan) and the western and eastern margins of North America^1,2^. Cypresses are characterized by their longevity and rot-resistant timbers, which are widely used in the construction of palaces, temples, and shrines as well as in furniture making. In Taiwan, two endemic cypress taxa, *Chamaecyparis formosensis* and *C. obtusa* var. *formosana*, are ecologically and economically important. They are montane conifers dominating cloud forests at 1500–2500 m above sea level^3^. Unfortunately, the IUCN red list regards them as vulnerable or endangered species^4^. It was estimated that ~ 60% of Taiwan’s cypress forests were logged during the early twentieth century^5^. Although logging of any primary forests is now prohibited in Taiwan, illegal logging is frequently reported and seriously damages not only the cypress populations but also the entire ecosystem of the cloud forest, where many stout and tall cypresses have survived for several thousand years.

**Figure 1:**
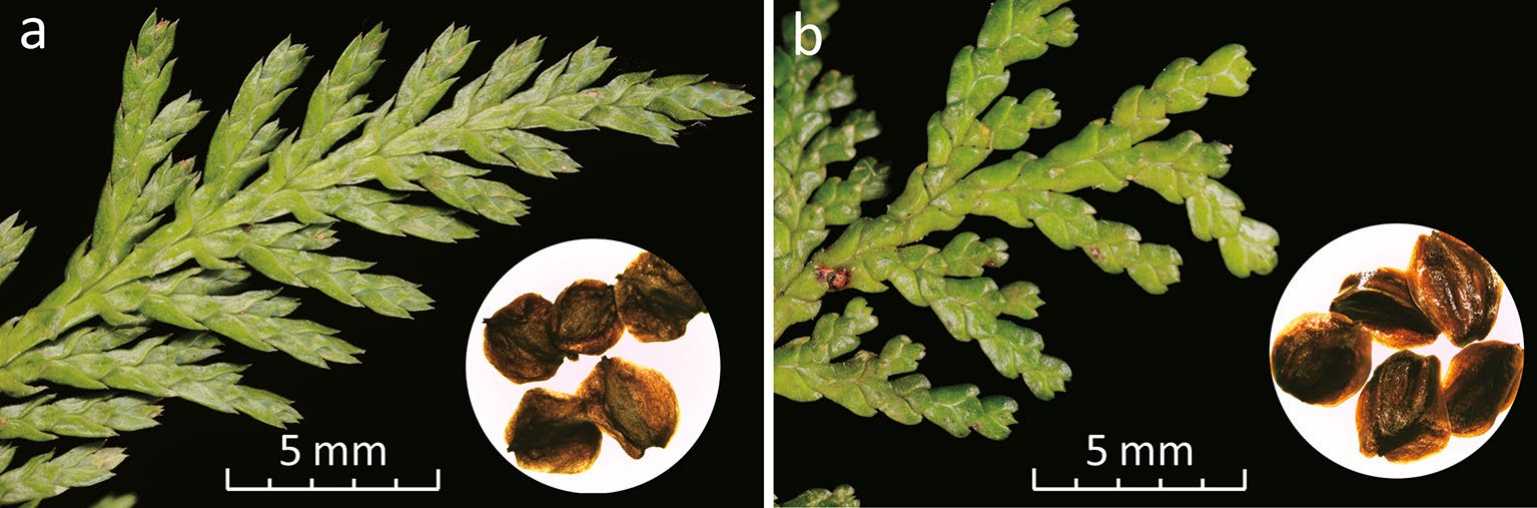
Photos of adult-leaves and winged seeds of *C. formosensis* (**a**) and *C. obtusa* var. *formosana* (**b**). The scale-bar unit is 1 mm.

Precise identification of seeds, seedlings, and timbers is vital for reforestation, and provides scientific support for prosecuting illegal logging crimes. Adult leaf tips can be used to distinguish *C. formosensis* from *C. obtusa* var. *formosana*, as the former’s are sharply pointed and the latter’s are abruptly acute (Fig. 1). In contrast, the winged seeds of both taxa are highly similar in size and morphology (Fig. 1), making it difficult to separate their seeds for managed reforestation. Timber identification based on wood anatomy relies on professional knowledge and practices^6,7^. Moreover, it is difficult to reliably identify species based on wood anatomical analyses^8^. In contrast, genetic approaches, such as DNA barcoding, offer an alternative and more straightforward line of evidence for timber identification^9,10^. Therefore, a comprehensive set of DNA references should be established prior to reliable identification^11,12^.

DNA barcoding, which generally requires a small and recoverable DNA segment, has recently attracted much attention in the fields of systematics, ecology, bio-conservation, and forensic investigation^13^. For example, plastid *rbcL* and *matK* were designated as core barcodes of land plants^14^, while the plastid *trnH-psbA* and nuclear ITS loci were suggested to be supplementary to these core barcodes^15,16^. Molecular technology provides authentic genetic information for species identification without requiring morphological characters^17^. However, none of the above-mentioned loci has ever been used to identify cypress species.

With high-throughput NGS data and advances in genomic assembly and analytical tools, utilization of the entire plastome or 35S rDNA sequence as a super-barcode was previously proposed to be a cost-effective way to discriminate species and evaluate phylogenies^18–20^. This super-barcoding approach is also encouraged to avoid problems of primer specificity, PCR amplification rate, and loss or duplication of the corresponding loci^21^. In addition, deciphering plastomes allows researchers to exploit hypervariable loci and lineage-specific indels, which have been demonstrated to be very efficient at discriminating orchid^22,23^ and yew^24^ species, respectively.

Despite the ecological and economic importance of cypresses, their DNA barcodes and associated references have not been investigated or established. In this paper, we address two questions: (1) Can we use whole plastomes and 35S rDNA as effective super-barcodes for identifying cypress species and varieties? and (2) If yes, then do they contain hypervariable loci that can be used as promising barcodes? To address these questions, we sampled 16 *C. formosensis* individuals and 10 *C. obtusa* var. *formosana* individuals from natural populations scattered across the cloud forests of Taiwan. Six cultivated individuals of another *C. obtusa* variety, *C. obtusa* var. *obtusa*, were also sampled from two remote localities. Sampling of multiple individuals per species/variety allowed us to evaluate identification rates based on the degree of species/variety-level monophyly in tree methods. We reconstructed both plastomes and 35S rDNAs from shallowly sequenced total DNA of the sampled cypresses using an approach known as genome skimming^25^. Our results reveal that the entire sequences of plastomes or 35S rDNAs not only serve as an effective super-barcode, but also contain short hypervariable loci and long lineage-specific indels that together constitute an alternative and powerful barcode system for cypress identification at both interspecies and inter-variety levels.

## Results

### Characteristics of cypress plastomes and nuclear 35S rDNAs

**Figure 2:**
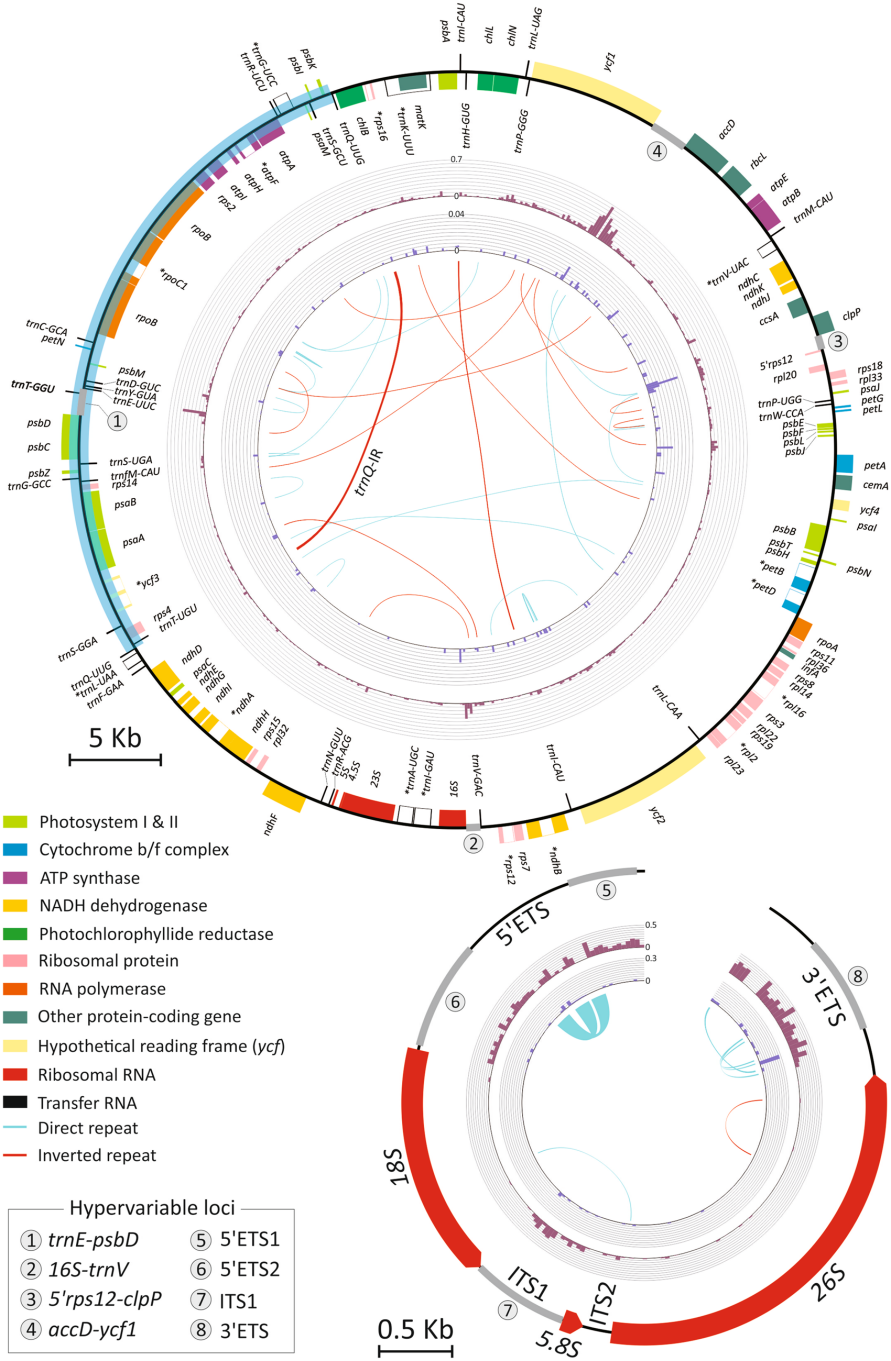
Circular maps of *Chamaecyparis* plastomes (above closed circle) and nuclear 35S rDNA (below open circle). Average inter-species and inter-variety pairwise substitution rates are depicted by histograms with gray-rose and steel-cyan colors, respectively. Repeat pairs are linked with lines. The region highlighted with blue is inverted in *C. hodginsii* and *C. lawsoniana*. A transfer RNA gene, *trnT-GGU* (bold), is uniquely missing in *C. formosensis*. Eight hypervariable loci are denoted by thick grey bars and Arabic numerals.

The plastomes of *C. formosensis*, *C. obtusa* var. *formosana,* and *C. obtusa* var. *obtusa* are GC-poor and mapped as circular molecules. Their lengths range from 126.8 to 127.8 kb (Table 1). Similar to *C. hodginsii* and *C. lawsoniana*, they lack the canonical repeat pair usually present in other seed plant plastomes. Notably, all cypress plastomes elucidated so far share a *trnQ*-containing repeat, which was previously called "*trnQ*-IR" in other cupressaceous species^26–28^. An approximately 20-kb region flanked by *trnQ*-IRs is inverted in *C. hodginsii* and *C. lawsoniana* compared to *C. formosensis* and *C. obtusa* varieties (Fig. 2). This suggests that the *trnQ*-IR remains active to trigger homologous recombination and generation of plastomic inversions in these cypresses. The plastid gene number is slightly variable among cypresses. For example, *trnT-GGU* is only missing in *C. formosensis* (Fig. 2). As a result, the absence of *trnT-GGU* readily distinguishes *C. formosensis* from other cypresses.

**Table 1:** Characteristics of plastomes and nuclear 35S rDNAs in *Chamaecyparis*.

Taxon	*C. formosensis*	*C. obtusa*^1^	*C. hodginsii*	*C. lawsoniana*
**Plastome**				
Size (Kb)	126.8‒127.2	127.3‒127.6	127.8	127.1
No. of genes	119	120	120	120
GC content (%)	35.0	35.0	35.0	35.0
**Nuclear 35S rDNA**^**2**^				
Size (Kb)	9.5‒10.1	8.9‒9.5	NA^3^	NA
No. of genes	3	3	NA	NA
GC content (%)	52.9‒53.0	53.0‒53.2	NA	NA

^1^Including *C. obtusa* var. *obtusa* and *C. obtusa* var. *formosana*; ^2^Including 5′ETS and 3′ETS; ^3^NA: not available.

The nuclear 35S rDNA sequences of the sampled 32 cypresses were assembled into linear molecules encompassing seven known genetic loci in the following order: 5′ETS, *18S*, ITS1, *5.8S*, ITS2, *26S*, and 3′ETS (Fig. 2). These 35S rDNAs are 8.9‒10.1 kb long, with GC contents over 50% (Table 1).

### Plastomes and 35S rDNAs as effective super-barcodes

**Figure 3:**
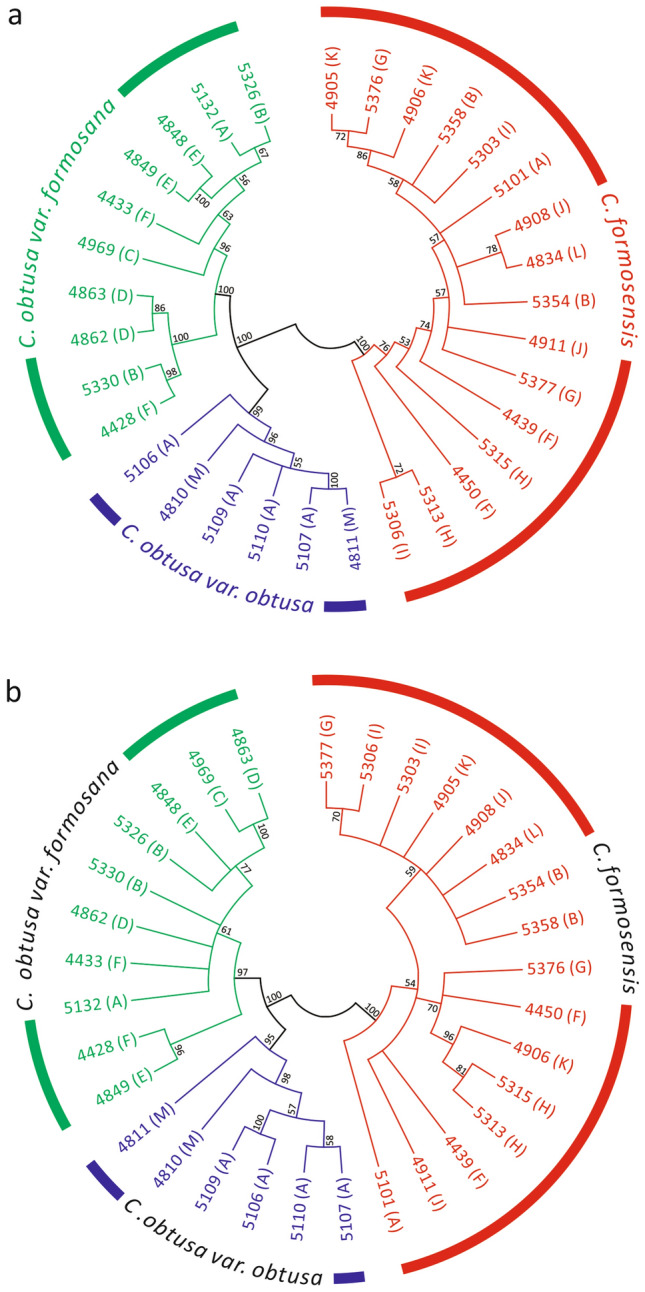
ML trees inferred from the entire plastome (**a**) and 35S rDNA (**b**) sequences. The trees are condensed under the 50% majority-rule consensus. Bootstrap values estimated from 1000 pseudo-replicates are shown at the nodes. Taxa are indicated by their voucher numbers, and population localities are denoted by letters within parentheses.

We evaluated the entire plastomes and 35S rDNA sequences to identify cypresses using maximum-likelihood tree approaches under the 50% majority-rule consensus. The plastome- and 35S rDNA-based trees share three monophyletic groups, each comprising individuals from the same species or varieties (Fig. 3). As a result, both plastome and 35S rDNA sequences achieved 100% identification rates at both inter-species and inter-variety levels.

We sampled one to two individuals from nine and six natural populations of *C. formosensis* and *C. obtusa* var. *formosana*, respectively (Supplementary Fig. 1). A 100% identification rate was not achieved at the inter-population level because individuals in the same population did not always form monophyletic groups in the plastome- (Fig. 3a) and 35S rDNA-based (Fig. 3b) trees. We did not estimate the inter-population identification rate of *C. obtusa var. obtusa* because the specimens were collected from reforestation areas or botanic gardens (Supplementary Table 1). Collectively, our results indicate that the entire sequences of both plastomes and 35S rDNAs are excellent super-barcodes capable of effectively identifying species and varieties, but not populations, of cypress.

### Nucleotide substitution rates are heterogeneous across plastomes and 35S rDNAs

Our sliding window analyses across the plastomes show that plastid nucleotide substitution rates (NSRs) vary from 0 to 0.667 and 0 to 0.036 substitutions per site at the inter-species and inter-variety levels, respectively (Fig. 2; Supplementary file 1). Variations in inter-species and inter-variety NSRs in the 35S rDNAs are 0‒0.483 and 0‒0.278 substitutions per site, respectively (Fig. 2; Supplementary file 1). These data highlight the fact that cypress plastomes and 35S rDNAs contain heterogeneous NSRs at both inter-species and inter-variety levels. We noted that the degree of plastid NSR heterogeneity dropped drastically at the inter-variety level. As a consequence, the 35 rDNAs are 7.72 times higher than plastomes in terms of the loci containing the highest inter-variety NSRs. Therefore, in cypresses, 35S rDNAs are likely more vulnerable to mutations than plastomes at the inter-variety level.

**Figure 4:**
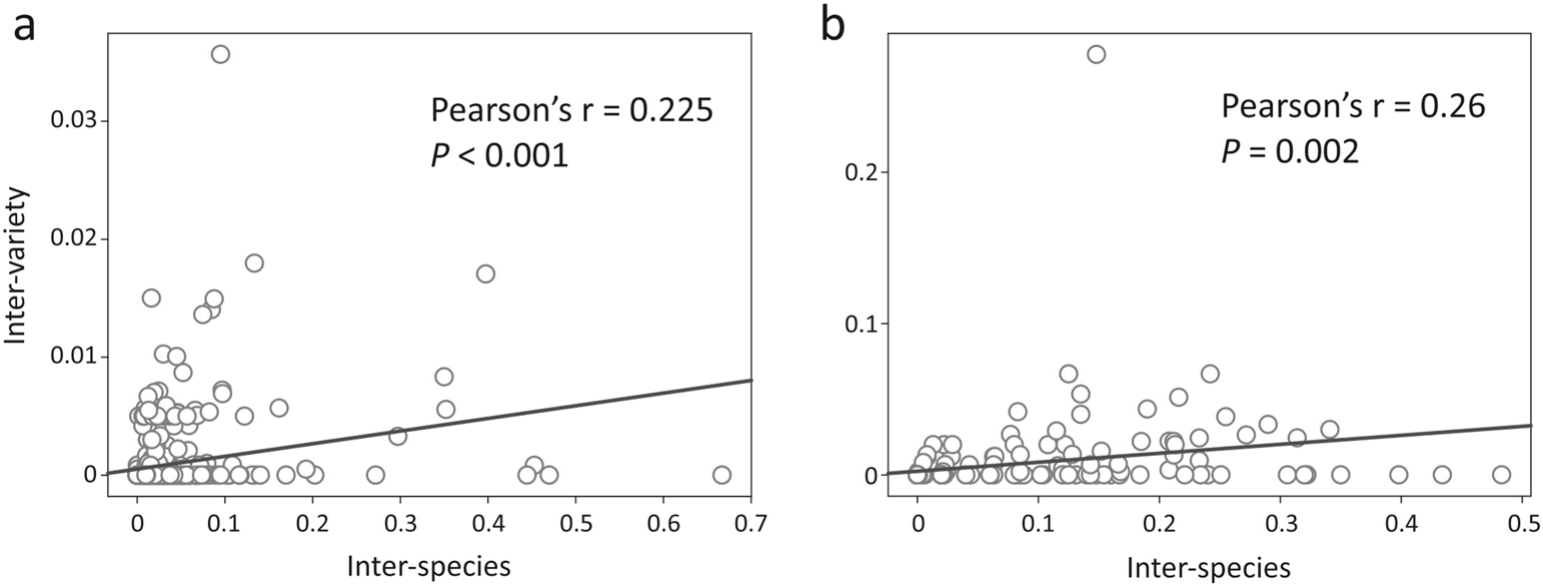
Estimated relationships between inter-species and inter-variety pairwise NSRs in the cypress plastomes (**a**) and 35S rDNAs (**b**).

Inter-species and inter-variety NSRs estimated from each sliding window were also compared to assess their relationships. We obtained 0.225 and 0.26 of Pearson’s correlation coefficients for the plastomes (Fig. 4a) and 35S rDNAs (Fig. 4b), respectively. This weak correlation suggests that the NSR evolution is not strongly linked between the inter-species and inter-variety levels, and that specific hypervariable loci may be required to identify cypresses at different taxonomic levels.

### ITS1 is a very promising barcode

Eight hypervariable loci were identified based on the estimated NSRs (Fig. 2). Seven of them (5′ETS1, 5′ETS2, 3′ETS, ITS1, *accD-ycf1*, *trnE-psbD*, and *16S-trnV*) feature high inter-species NSRs, while the last one (*5′rps12-clpP*) has a relatively higher inter-variety NSR than other plastid loci. These loci are all noncoding regions that range from 524 to 1,835 bp in length (Table 2). We separated the 5′ETS region into 5′ETS1 and 5′ETS2 loci to exclude long repeats inside the loci (Fig. 2). We also included four loci that were previously reported to be promising barcodes. For example, the locus of *trnH-psbA* is a widely acceptable barcode in diverse plant lineages^29–31^. Two loci, *matK* and *rbcL*, constitute the core barcodes for land plants^14^. Meanwhile, the locus of *accD* was reported to be a promising barcode that can be used with the plastome super-barcode to identify yews^24^. Table 2 demonstrates that all 12 examined loci have a clear barcoding gap at the inter-species level because there is no overlap between inter-species and intra-species NSRs. In contrast, inter-variety barcoding gaps are observed only in the loci of 5′ETS1, ITS1, *trnH-psbA*, and *rbcL*.

**Table 2:** Nuclear 35S rDNA and plastomic loci examined for identification of cypresses species and varieties.

Locus	Type	Length (bp)	Pairwise distance (substitutions per site)				BS value (%) for monophyly		
			Inter-species	Intra-species	Inter-variety	Intra-variety	CF^1^	CVF	CVO
5′ETS1	Nucleus	511‒514	0.123‒0.131	0‒0.006	0.004‒0.006	0‒0.002	99	ND^2^	88
5′ETS2	Nucleus	920‒938	0.092‒0.1	0‒0.01	0.004‒0.01	0‒0.004	99	ND	96
3′ETS	Nucleus	524‒816	0.196‒0.234	0‒0.05	0.011‒0.05	0‒0.038	99	65	79
ITS1	Nucleus	733‒734	0.114‒0.118	0‒0.014	0.008‒0.014	0‒0.005	99	98	79
*accD-ycf1*	Plastome	1375‒1835	0.308‒0.33	0‒0.03	0.004‒0.01	0‒0.005	100	70	ND
*trnE-psbD*	Plastome	1290‒1327	0.069‒0.073	0‒0.001	0‒0.001	0‒0.001	99	ND	ND
*16S-trnV*	Plastome	548‒773	0.159‒0.168	0‒0.002	0‒0.002	0‒0.002	99	ND	86
*5′rps12-clpP*	Plastome	1057‒1189	0.065‒0.085	0‒0.042	0.003‒0.032	0.001‒0.042	99	ND	ND
***trnH-psbA***^3^	Plastome	438‒461	0.036‒0.038	0‒0.002	0.002	0	99	63	63
***matK***	Plastome	1530‒1533	0.012‒0.014	0‒0.001	0.001	0‒0.001	99	61	ND
***rbcL***	Plastome	1428	0.006‒0.007	0‒0.001	0.001	0	99	67	ND
***accD***	Plastome	2274‒2277	0.039‒0.04	0‒0.002	0.001‒0.002	0‒0.001	99	67	87

^1^CF: *C. formosensis*; CVF: *C. obtusa* var. *formosana*; CVO: *C. obtusa* var. *obtusa*; ^2^ND: not detected; ^3^Loci highlighted in bold are promising markers proposed in previous studies.

Unrooted neighbor-joining trees inferred from each of the 12 loci were generated to identify the cypresses at both inter-species and inter-variety levels. Under the 50% majority-rule consensus, these 12 loci congruently suggest that all sampled *C. formosensis* individuals constitute a monophyletic group with strong bootstrap values ranging from 99 to 100% (Table 2). However, only the 3′ETS, ITS1, *trnH-psbA*, and *accD* loci successfully resolved the two *C. obtusa* varieties as separate monophyletic groups. When the bootstrap values, locus lengths, and existence of barcoding gaps are taken into account, ITS1 appears to be the most promising barcode capable of effectively identifying both cypress species and varieties.

### Long indels for species discrimination

**Figure 5:**
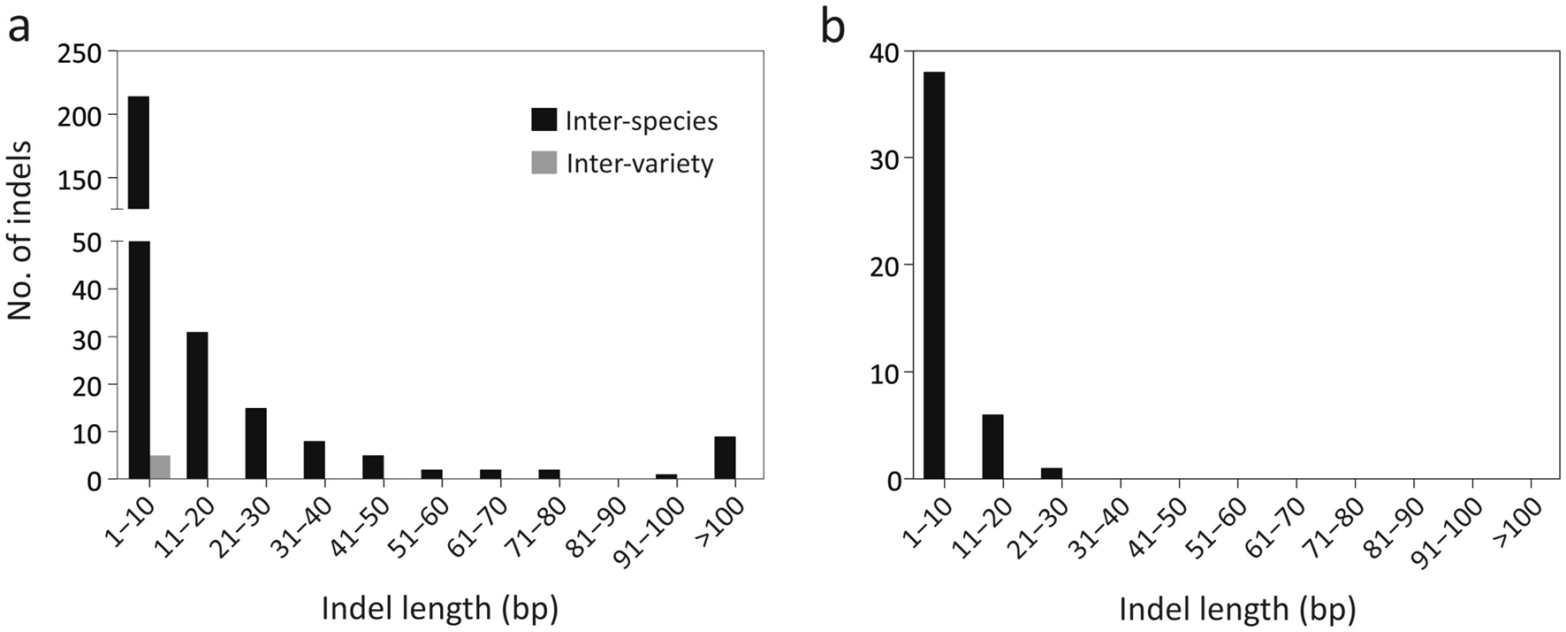
Summary of species-specific (black bar) and variety-specific (grey bar) indels found in the cypress plastomes (**a**) and 35S rDNAs (**b**).

Lineage-specific indels are a straightforward tool for distinguishing species. We identified five and 287 plastid indels as variety- and species-specific, respectively (Supplementary Table 2). These indels occur mostly in intergenic spaces (76.7%), followed by coding regions (15.4%) and introns (7.9%). In addition, all variety-specific indels are located in the plastid intergenic spaces except for one, which is located in the *ycf1* gene. Although the majority (74.4%) of the plastid indels are shorter than or equal to 10 bp, nine species-specific indels are longer than 100 bp (Fig. 5a). These long plastid indels are useful markers for separating *C. formosensis* from *C. obtusa* varieties because they are readily detectable using PCR gel electrophoresis.

We detected 45 indels in 35S rDNAs. They are species-specific, shorter than 100 bp (Table S3; Fig. 5b; Supplementary Table 3), and located in the 3′ETS (51.1%), 5′ETS (40%), ITS1 (4.4%), and ITS2 (4.4%).

## Discussion

We obtained 3.03‒3.76 Gb of NGS sequences for each of the 32 sampled cypresses (Supplementary Table 1). Our assemblies of the complete plastomes and full-length 35S rDNA sequences suggest that genome skimming is a cost-effective and robust strategy for assembling the two sequences in cypresses, whose genomes are huge (estimated to be 17.1‒18.6 pg/2C)^32^. We were not able to recover high-quality mitochondrial contigs from NGS reads, perhaps because (1) the copy number of mitochondrial DNAs is relatively low^33,34^ and (2) seed plant mitochondria frequently undergo inter- and intra-genomic recombination, generating a complicated set of subgenomes^35–37^. Nonetheless, our study clearly demonstrates that both the plastome and 35S rDNA are valuable resources for developing effective barcodes to identify cypress species and varieties.

Previous studies have used entire plastome sequences to identify various seed plant species, and argued that plastomes are effective super-barcodes^20,23,24,38,39^. Our analysis further confirms that entire plastomes are effective super-barcodes for identifying both cypress species and varieties (Fig. 3a). In addition, using 35S rDNAs as super-barcodes also yielded a 100% identification rate at both the inter-species and inter-variety levels (Fig. 3b). This finding further suggests that the 35S rDNAs are more cost-effective super-barcodes than plastomes since the former is approximately 13 times shorter than the latter in the cypresses (Table 1).

Plastomic rearrangements between congeneric species, albeit rare in seed plants, have been documented in some lineages, such as *Pelargonium* and *Hypseocharis*^40^, *Podocarpus*^41^, *Juniperus*^26^, *Calocedrus*^28^, and *Halamphora*^42^. Recently, plastomic rearrangements were found in conspecific individuals of yews, likely due to recurrent shifts between predominant and substoichiometric isomeric plastomes^24^. In this study, we detected a 23-kb plastomic inversion between cypress species (Fig. 2). This non-collinear genome organization impedes the performance of entire plastome alignments, thus decreasing the feasibility of utilizing plastomes as super-barcodes. In contrast, the component and organization of 35S rDNAs are highly uniform across the land plant kingdom^43^, thus making it a good candidate for DNA barcoding outside of cypresses too.

However, using plastomes and 35S rDNAs as super-barcodes has some limitations. First, the assembly of plastomes and 35S rDNAs from NGS reads is labor-intensive, including DNA extraction, library construction, sequencing, quality trimming, assembly, and annotation. Second, plastid DNA copies are highly variable among lineages, tissues, developmental stages, and even growth conditions^44^, making it tough to standardize the amount of NGS reads required to assemble plastomes. Similarly, 35S rDNA copy numbers vary greatly between species^43^. Third, NGS requires a larger amount of DNA than PCR-based methods, and extracting sufficient DNA from woody materials may be challenging when DNA is rare or degraded^10^.

Although concatenation of multiple loci is frequently adopted in plant DNA barcoding^21^, we demonstrate that four loci—3′ETS, ITS1, *trnH-psbA*, and *accD*—are individually capable of delimiting the cypress species and varieties (Table 2). In cupressophytes, however, *accD* is generally elongated and longer than 2 kb, with high lineage-specific repeat content^45,46^. A gene of this size requires at least four runs of Sanger sequencing to sequence the full gene. Moreover, an inverse relationship between amplicon lengths and recoverable rates was observed when PCR templates were the DNA extracted from wood^10,47^. As cypresses are threatened by illegal logging because of their valuable timber, *accD* may not be a suitable barcoding locus for forensic timber identification. Therefore, we screened nine species-specific indels > 100 bp long (Fig. 5; Supplementary Table 3). They can serve as supplementary markers that better discriminate among cypress species.

In conclusion, we successfully obtained entire plastomes and 35S rDNAs from a shallow sequencing of all sampled cypress species and varieties. Both plastomes and 35S rDNAs have a strong potential to be effective super-barcodes for identifying cypress species and varieties. We also identified three loci (3′ETS, ITS1, and *trnH-psbA*) with appropriate lengths, high NSRs, and 100% identification rates at both the inter-species and inter-variety levels. These loci, together with the nine supplementary indel markers, can be used in an alternative barcode system when the botanical specimen has poor or low DNA content. Our new sequence data and innovative barcode systems not only enrich the availability of genetic references for cypresses, but also contribute to their conservation, authentication, reforestation, and forensic timber identification to stop illegal logging and its associated trade.

## Materials and methods

### Sample collection

We sampled 26 endemic cypress individuals—16 *C. formosensis* and 10 *C. obtusa* var. *formosana*—from cloud forests in the Central Mountain Range of Taiwan (Supplementary Fig. 1), with 1‒2 individuals sampled in each population. In addition, six cultivated individuals of *C. obtusa* var. *obtusa* were also sampled from two remote localities (four and two from localities A and B, respectively; Supplementary Table 1). These closely related taxa were identified based on their morphologies (Fig. 1) and planting histories. All vouchers and the associated DNAs are deposited in the germplasm bank of the Ministry of Justice Investigation Bureau (MJIB), New Taipei City, Taiwan.

### DNA extraction, sequencing, and assembling

Total genomic DNA was extracted from 2 g of fresh leaves using a modified CTAB method^48^ with 0.1% of polyvinylpyrrolidone (PVP-40, Sigma) incorporated into the extraction buffer. The extracted DNA was sheared into fragments 400‒600 bp long, and DNA libraries were constructed using Ovation Rapid Library Preparation kits (NuGEN). Each library was sequenced on an Illumina HiSeq 4000 platform in Tri-I Biotech Company (New Taipei City, Taiwan) to generate 16.7‒46.26 million 2 × 150 bp pair-end reads that amassed a total of 3.03‒7.36 Gb (Supplementary Table 1). After removing adapters and non-qualified bases using Trimmomatic 0.38^49^, these reads were *de-novo* assembled with kmer lengths = 21, 33, 55, 77, and 91 in SPAdes 3.14.0^50^. Plastomic and 35S rDNA contigs were searched using BLAST + 2.10.0^51^ with the plastome (NC034943) and ITS (AY211258) of *C. formosensis* as references. Gaps between plastomic contigs were closed using GapCloser v1.12^52^. We used SEQuel v1.0.2^53^ to do base-scale corrections of the assembled plastomes and 35S rDNAs.

### Plastome and 35S rDNA annotation

Plastome annotation was performed using GeSeq^54^ with the default settings for search identities. For 35S rDNA, the 18S, 5.8S, and 26S ribosomal RNAs were predicted on RNAmmer 1.2 Server^55^. The annotated genes were further adjusted manually based on alignments of their orthologous genes from other cupressaceous species. Plastomes and 35S rDNAs were visualized using Circos 0.67^56^.

### Sequence alignment and phylogenetic tree construction

Plastomes and 35S rDNAs were aligned using MAFFT 7.074^57^ with the algorithm = auto, scoring matrix = 200 PAM/k = 2, gap open penalty = 1.53, and offset value = 0.123. Maximum likelihood (ML) trees inferred from these alignments were estimated under a GTRGAMMAI model and 1,000 bootstrap replicates in RAxML v8.2^58^. Neighbor-joining (NJ) trees based on single loci were constructed with a P-distance model, uniform rates among sites, and 1000 bootstrap replicates in MEGA X 10.1.759^59^. All yielded trees were condensed under the 50% majority-rule consensus.

### Exploration of plastomic inversions

We used progressiveMauve^60^ to estimate plastomic inversions among *C. formosensis*, *C. hodginsii*, *C. lawsoniana*, *C. obtusa* var. *formosana*, and *C. obtusa* var. *obtusa*.

### Calculation of nucleotide substitution rates

The average number of nucleotide substitutions per site between species or varieties was calculated to explore the hypervariable loci using the non-overlapping sliding window approaches in DnaSP v6^61^. The window length was set to be 200 and 50 bp for plastomes and 35S rDNAs, respectively. To investigate barcoding gaps, sequences of the examined loci were aligned using MUSCLE 3.8.31^62^, followed by an estimate of pairwise nucleotide substitution rates (NSRs) using MEGA X with a P-distance model and uniform rates among sites.

### Detection of repeats and indels

NCBI blastn was employed to compare each plastome or 35S rDNA against itself with the default settings. We discarded the matched pairs with sequence identities less than 90%. The remaining pairs of repeats were further checked manually to remove redundancies. Indels between species or varieties were detected using DnaSP.

## Supplementary information

Supplementary Information.

Supplementary Information.

## Data Availability

All DNA sequences were deposited into DDBJ DataBank under accession numbers LC516824‒LC529365 (Supplementary Table 1).
