# Supplementary material for: Genome skimming and exploration of DNA barcodes for Taiwan endemic cypresses
Source: Sci Rep. 2020 Nov 26;10:20650. doi: 10.1038/s41598-020-77492-2 (PMC7693304; doi:10.1038/s41598-020-77492-2)
Supplement: Supplementary file 2 — Supplementary Information. [file 41598_2020_77492_MOESM2_ESM.pdf]

Genome skimming and exploration of DNA barcodes for Taiwan endemic cypresses

Chung-Shien Wu[1], Edi Sudianto[1], Yu-Mei Hung[2], Bo-Cyun Wang[1], Chiun-Jr Huang[3], Chi-Tsong Chen[2,*], Shu-Miaw Chaw[1,*]

1. Biodiversity Research Center, Academia Sinica, Taipei 11529, Taiwan.
2. Department of Forensic Science Investigation Bureau, Ministry of Justice, New Taipei City 231209, Taiwan.
3. School of Forestry and Resource Conservation, National Taiwan University, Taipei 10617, Taiwan.

*Corresponding Authors: Chi-Tsong Chen (chen33039@gmail.com); Shu-Miaw Chaw (smchaw@sinica.edu.tw)

Supplementary Figure 1. Map of Taiwan showing the localities of sampled cypress populations (letters: A–L). Note that *C. formosensis* and *C. obtusa* varieties are sometimes mixed together, and thus labeled with the same letter. Two *C. obtusa* var. *obtusa* individuals (labeled "M" in Supplementary Table 1) are not included in the map because they were sampled from the Arnold Arboretum of Harvard University. The map was generated using QGIS v2.14.0 (https://www.qgis.org/en/site/).

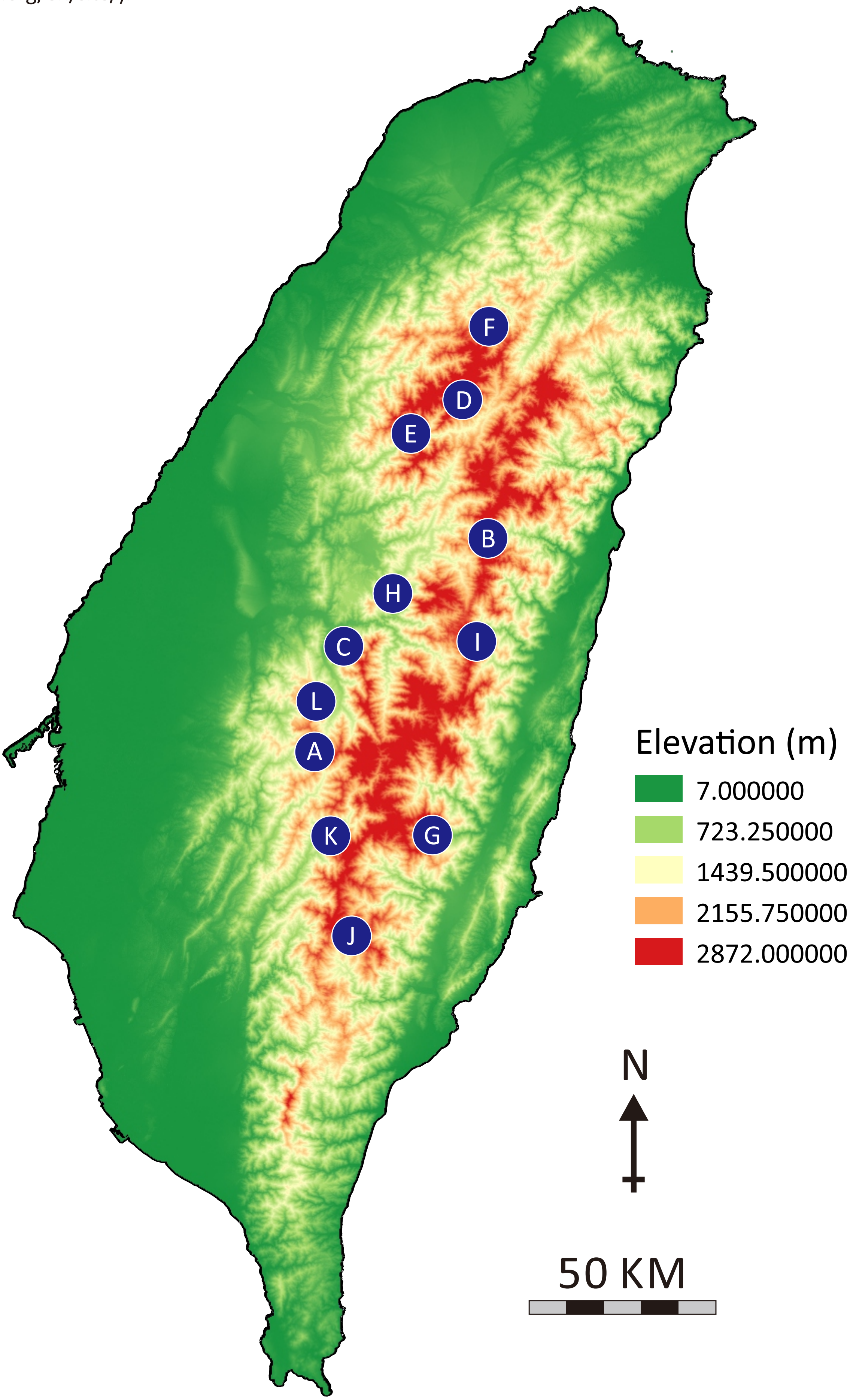

Supplementary Table 1. Summary of the sampled taxa and their voucher number, sampling locality, GenBank accession, and sequencing status. *Letters refer to the locality shown in Supplementary Figure 1 except for B, which indicates the Arnold Arboretum of Harvard University

Species	Voucher number	GenBank accession		Sampling locality*	Total pair-end reads (million)	Total sequenced bases (Gb)
		Plastome	35S rDNA			
Chamaecyparis formosensis	5354	LC529343	LC518083	C	17.25	3.17
Chamaecyparis formosensis	5303	LC522363	LC518087	J	18.26	3.37
Chamaecyparis formosensis	4911	LC522361	LC518082	K	18.59	3.25
Chamaecyparis formosensis	4439	LC522086	LC516824	G	19.51	3.06
Chamaecyparis formosensis	5376	LC529344	LC518089	H	19.56	3.59
Chamaecyparis formosensis	5315	LC529345	LC518080	I	20.1	3.68
Chamaecyparis formosensis	5101	LC522362	LC518093	A	20.39	3.73
Chamaecyparis formosensis	4905	LC522088	LC518091	L	20.4	3.6
Chamaecyparis formosensis	5306	LC522364	LC518088	J	20.67	3.81
Chamaecyparis formosensis	4906	LC522359	LC518092	L	21.5	3.76
Chamaecyparis formosensis	5358	LC529346	LC518084	C	21.88	4.03
Chamaecyparis formosensis	5377	LC529347	LC518090	H	22.08	4.07
Chamaecyparis formosensis	4450	LC522087	LC518085	G	23.03	3.83
Chamaecyparis formosensis	4834	LC529348	LC518086	M	23.3	4.14
Chamaecyparis formosensis	4908	LC522360	LC518081	K	23.92	4.26
Chamaecyparis formosensis	5313	LC529349	LC518079	I	24.82	4.56
Chamaecyparis obtusa var. formosana	4428	LC529350	LC518101	G	18.67	3.03
Chamaecyparis obtusa var. formosana	5326	LC529351	LC518099	C	19.17	3.49
Chamaecyparis obtusa var. formosana	5132	LC529352	LC518103	A	19.97	3.71

Chamaecyparis obtusa var. formosana	4848	LC529353	LC518096	F	20.13	3.26
Chamaecyparis obtusa var. formosana	4862	LC529354	LC518094	E	20.42	3.58
Chamaecyparis obtusa var. formosana	4849	LC529355	LC518097	F	20.81	3.65
Chamaecyparis obtusa var. formosana	4969	LC529356	LC518098	D	20.81	3.72
Chamaecyparis obtusa var. formosana	5330	LC529357	LC518100	C	20.83	3.84
Chamaecyparis obtusa var. formosana	4863	LC529358	LC518095	E	20.9	3.69
Chamaecyparis obtusa var. formosana	4433	LC529359	LC518102	G	23.02	3.75
Chamaecyparis obtusa var. obtusa	5106	LC529362	LC518108	A	16.7	3.09
Chamaecyparis obtusa var. obtusa	5107	LC529363	LC518109	A	18.1	3.34
Chamaecyparis obtusa var. obtusa	5109	LC529364	LC518104	A	18.1	3.34
Chamaecyparis obtusa var. obtusa	5110	LC529365	LC518105	A	43.48	6.77
Chamaecyparis obtusa var. obtusa	4810	LC529360	LC518106	B	43.75	6.84
Chamaecyparis obtusa var. obtusa	4811	LC529361	LC518107	B	46.26	7.36

Supplementary Table 2. Plastomic indels found to discriminate *Chamaecyparis* species or varieties			
Indel ID	Indel length (bp)	Indel type	Location
Indel_1	5	Inter-species	*psbA-trnK*
Indel_2	9	Inter-species	*psbA-trnK*
Indel_3	5	Inter-species	*psbA-trnK*
Indel_4	3	Inter-species	*matK*
Indel_5	9	Inter-species	*trnK* intron
Indel_6	5	Inter-species	*trnK* intron
Indel_7	4	Inter-species	*trnK* intron
Indel_8	11	Inter-species	*trnK* intron
Indel_9	4	Inter-species	*trnK-rps16*
Indel_10	4	Inter-species	*trnK-rps16*
Indel_11	2	Inter-species	*rps16-chlB*
Indel_12	6	Inter-species	*rps17-chlB*
Indel_13	3	Inter-species	*rps18-chlB*
Indel_14	3	Inter-species	*psbK-psbI*
Indel_15	5	Inter-species	*psbK-psbI*
Indel_16	3	Inter-species	*psbK-psbI*
Indel_17	41	Inter-species	*psbI-trnS*
Indel_18	1	Inter-species	*psaM-trnG*
Indel_19	4	Inter-species	*psaM-trnG*
Indel_20	26	Inter-species	*psaM-trnG*
Indel_21	2	Inter-species	*psaM-trnG*
Indel_22	5	Inter-species	*psaM-trnG*
Indel_23	1	Inter-species	*psaM-trnG*
Indel_24	1	Inter-species	*trnG-trnR*
Indel_25	9	Inter-species	*trnG-trnR*
Indel_26	3	Inter-species	*atpF-atpH*
Indel_27	11	Inter-species	*atpF-atpH*
Indel_28	1	Inter-species	*atpH-atpI*
Indel_29	3	Inter-species	*atpH-atpI*
Indel_30	4	Inter-variety	*atpH-atpI*
Indel_31	9	Inter-species	*atpH-atpI*
Indel_32	1	Inter-species	*atpI-rps2*
Indel_33	4	Inter-species	*rps2-rpoB*
Indel_34	327	Inter-species	*rpoC2*

Indel_35	6	Inter-species	*rpoC2*
Indel_36	8	Inter-species	*rpoC2-rpoC1*
Indel_37	1	Inter-species	*rpoC1* intron
Indel_38	4	Inter-species	*rpoC1* intron
Indel_39	3	Inter-species	*rpoB*
Indel_40	122	Inter-species	*rpoB-trnC*
Indel_41	36	Inter-species	*rpoB-trnC*
Indel_42	1	Inter-species	*trnC-petN*
Indel_43	3	Inter-species	*trnC-petN*
Indel_44	4	Inter-species	*petN-psbM*
Indel_45	5	Inter-species	*petN-psbM*
Indel_46	1	Inter-species	*petN-psbM*
Indel_47	56	Inter-species	*petN-psbM*
Indel_48	7	Inter-species	*petN-psbM*
Indel_49	103	Inter-species	*psbM-trnD*
Indel_50	80	Inter-species	*psbM-trnD*
Indel_51	5	Inter-species	*psbM-trnD*
Indel_52	5	Inter-species	*psbM-trnD*
Indel_53	10	Inter-species	*psbM-trnD*
Indel_54	17	Inter-species	*trnY-trnE*
Indel_55	42	Inter-species	*trnT-psbD*
Indel_56	46	Inter-species	*trnT-psbD*
Indel_57	27	Inter-species	*trnT-psbD*
Indel_58	100	Inter-species	*trnT-psbD*
Indel_59	4	Inter-species	*psbC-trnS*
Indel_60	5	Inter-species	*psbC-trnS*
Indel_61	2	Inter-species	*psbC-trnS*
Indel_62	6	Inter-species	*trnS-psbZ*
Indel_63	15	Inter-species	*trnS-psbZ*
Indel_64	10	Inter-species	*psbZ-trnG*
Indel_65	4	Inter-species	*psbZ-trnG*
Indel_66	12	Inter-species	*psbZ-trnG*
Indel_67	8	Inter-species	*psbZ-trnG*
Indel_68	22	Inter-species	*trnG-trnfM*
Indel_69	5	Inter-species	*rps14-psaB*
Indel_70	1	Inter-species	*psaA-ycf3*
Indel_71	19	Inter-species	*ycf3 intron*

Indel_72	1	Inter-species	*ycf3 intron*
Indel_73	4	Inter-species	*ycf3-trnS*
Indel_74	1	Inter-species	*ycf4-trnS*
Indel_75	9	Inter-species	*ycf5-trnS*
Indel_76	5	Inter-species	*ycf6-trnS*
Indel_77	8	Inter-species	*ycf7-trnS*
Indel_78	1	Inter-species	*ycf8-trnS*
Indel_79	1	Inter-species	*trnS-rps4*
Indel_80	12	Inter-species	*trnS-rps5*
Indel_81	9	Inter-species	*rps4-trnT*
Indel_82	6	Inter-species	*trnQ-trnL*
Indel_83	10	Inter-species	*trnL intron*
Indel_84	6	Inter-species	*trnL intron*
Indel_85	3	Inter-species	*trnL-trnF*
Indel_86	8	Inter-species	*trnL-trnF*
Indel_87	3	Inter-variety	*trnL-trnF*
Indel_88	1	Inter-species	*trnL-trnF*
Indel_89	8	Inter-species	*trnF-ndhD*
Indel_90	25	Inter-species	*trnF-ndhD*
Indel_91	1	Inter-species	*trnF-ndhD*
Indel_92	3	Inter-species	*trnF-ndhD*
Indel_93	1	Inter-species	*trnF-ndhD*
Indel_94	8	Inter-species	*psaC-ndhE*
Indel_95	4	Inter-species	*ndhE-ndhG*
Indel_96	22	Inter-species	*ndhG-ndhI*
Indel_97	18	Inter-species	*ndhA* intron
Indel_98	10	Inter-species	*ndhA* intron
Indel_99	4	Inter-species	*ndhA* intron
Indel_100	4	Inter-species	*ndhH-rps15*
Indel_101	4	Inter-species	*rps15-rpl32*
Indel_102	32	Inter-species	*rps15-rpl33*
Indel_103	3	Inter-species	*rpl32*
Indel_104	2	Inter-species	*rpl32-ndhF*
Indel_105	9	Inter-species	*ndhF*
Indel_106	3	Inter-species	*ndhF-trnN*
Indel_107	11	Inter-species	*ndhF-trnN*
Indel_108	4	Inter-species	*ndhF-trnN*

Indel_109	6	Inter-species	*ndhF-trnN*
Indel_110	6	Inter-species	*ndhF-trnN*
Indel_111	170	Inter-species	*trnN-trnR*
Indel_112	30	Inter-species	*trnN-trnR*
Indel_113	78	Inter-species	*trnN-trnR*
Indel_114	1	Inter-species	*trnN-trnR*
Indel_115	42	Inter-species	*trnR-5S*
Indel_116	23	Inter-species	*5S-4.5S*
Indel_117	105	Inter-species	*23S*
Indel_118	6	Inter-species	*23S*
Indel_119	4	Inter-species	*23S*
Indel_120	5	Inter-species	*trnI* intron
Indel_121	1	Inter-species	*trnI-16S*
Indel_122	15	Inter-species	*16S-trnV*
Indel_123	2	Inter-species	*16S-trnV*
Indel_124	103	Inter-species	*16S-trnV*
Indel_125	1	Inter-species	*16S-trnV*
Indel_126	8	Inter-variety	*16S-trnV*
Indel_127	5	Inter-species	*16S-trnV*
Indel_128	1	Inter-species	*16S-trnV*
Indel_129	26	Inter-species	*16S-trnV*
Indel_130	2	Inter-species	*16S-trnV*
Indel_131	8	Inter-species	*trnV*
Indel_132	6	Inter-species	*trnV-3'rps12*
Indel_133	1	Inter-species	*trnV-3'rps12*
Indel_134	5	Inter-species	*trnV-3'rps12*
Indel_135	4	Inter-species	*trnV-3'rps12*
Indel_136	6	Inter-species	*trnV-3'rps12*
Indel_137	1	Inter-species	*trnV-3'rps12*
Indel_138	18	Inter-species	*trnV-3'rps12*
Indel_139	1	Inter-species	*3'rps12* intron
Indel_140	5	Inter-species	*3'rps12* intron
Indel_141	11	Inter-species	*ndhB* intron
Indel_142	9	Inter-species	*ndhB* intron
Indel_143	5	Inter-species	*ndhB* intron
Indel_144	17	Inter-species	*ndhB-trnI*
Indel_145	8	Inter-species	*ndhB-trnI*

Indel_146	10	Inter-species	*trnI-ycf2*
Indel_147	3	Inter-species	*trnI-ycf2*
Indel_148	9	Inter-species	*trnI-ycf2*
Indel_149	11	Inter-species	*ycf2*
Indel_150	2	Inter-species	*ycf2*
Indel_151	2	Inter-species	*ycf2*
Indel_152	6	Inter-species	*ycf2*
Indel_153	6	Inter-species	*ycf2*
Indel_154	24	Inter-species	*ycf2*
Indel_155	6	Inter-species	*ycf2*
Indel_156	33	Inter-species	*ycf2*
Indel_157	39	Inter-species	*ycf2*
Indel_158	9	Inter-species	*ycf2*
Indel_159	9	Inter-species	*ycf2*
Indel_160	3	Inter-species	*ycf2*
Indel_161	6	Inter-species	*ycf2*
Indel_162	6	Inter-species	*ycf2*
Indel_163	3	Inter-species	*ycf2*
Indel_164	6	Inter-species	*ycf2*
Indel_165	18	Inter-species	*ycf2-trnL*
Indel_166	5	Inter-species	*ycf2-trnL*
Indel_167	4	Inter-species	*ycf2-trnL*
Indel_168	4	Inter-species	*trnL-rpl23*
Indel_169	15	Inter-species	*trnL-rpl23*
Indel_170	8	Inter-species	*rpl22-rps3*
Indel_171	8	Inter-species	*rpl16* intron
Indel_172	10	Inter-species	*rpl16* intron
Indel_173	7	Inter-species	*rps8-infA*
Indel_174	7	Inter-species	*rps8-infA*
Indel_175	5	Inter-species	*rps8-infA*
Indel_176	1	Inter-species	*rpl36-rps11*
Indel_177	20	Inter-species	*rpl36-rps11*
Indel_178	5	Inter-species	*rpoA*
Indel_179	1	Inter-species	*rpoA-petD*
Indel_180	5	Inter-species	*rpoA-petD*
Indel_181	1	Inter-species	*petD-petB*
Indel_182	5	Inter-species	*petD-petB*

Indel_183	4	Inter-species	*petD-petB*
Indel_184	1	Inter-species	*petB* intron
Indel_185	3	Inter-species	*petB* intron
Indel_186	4	Inter-species	*petB-psbH*
Indel_187	1	Inter-species	*psbN-psbT*
Indel_188	7	Inter-species	*psbB-psaI*
Indel_189	9	Inter-species	*psbB-psaI*
Indel_190	3	Inter-species	*psaI-ycf4*
Indel_191	5	Inter-species	*psaI-ycf4*
Indel_192	10	Inter-species	*psaI-ycf4*
Indel_193	67	Inter-species	*ycf4-cemA*
Indel_194	1	Inter-species	*ycf4-cemA*
Indel_195	1	Inter-species	*ycf4-cemA*
Indel_196	5	Inter-species	*ycf4-cemA*
Indel_197	2	Inter-species	*ycf4-cemA*
Indel_198	1	Inter-species	*ycf4-cemA*
Indel_199	1	Inter-species	*petA-psbJ*
Indel_200	5	Inter-species	*petA-psbJ*
Indel_201	6	Inter-species	*petA-psbJ*
Indel_202	39	Inter-species	*petA-psbJ*
Indel_203	5	Inter-species	*petA-psbJ*
Indel_204	1	Inter-species	*psbE-petL*
Indel_205	12	Inter-species	*petL-petG*
Indel_206	3	Inter-species	*trnW-trnP*
Indel_207	1	Inter-species	*trnW-trnP*
Indel_208	5	Inter-species	*trnW-trnP*
Indel_209	1	Inter-species	*5'rps12-clpP*
Indel_210	6	Inter-species	*5'rps12-clpP*
Indel_211	2	Inter-species	*5'rps12-clpP*
Indel_212	11	Inter-species	*5'rps12-clpP*
Indel_213	50	Inter-species	*5'rps12-clpP*
Indel_214	5	Inter-species	*5'rps12-clpP*
Indel_215	5	Inter-species	*5'rps12-clpP*
Indel_216	3	Inter-species	*5'rps12-clpP*
Indel_217	1	Inter-species	*clpP*
Indel_218	8	Inter-species	*clpP*
Indel_219	6	Inter-species	*clpP-ccsA*

Indel_220	33	Inter-species	*clpP-ccsA*
Indel_221	39	Inter-species	*clpP-ccsA*
Indel_222	6	Inter-species	*ccsA-ndhJ*
Indel_223	2	Inter-species	*ccsA-ndhJ*
Indel_224	20	Inter-species	*ccsA-ndhJ*
Indel_225	52	Inter-species	*ccsA-ndhJ*
Indel_226	124	Inter-species	*ccsA-ndhJ*
Indel_227	6	Inter-species	*ndhJ-ndhK*
Indel_228	9	Inter-species	*ndhK*
Indel_229	5	Inter-species	*ndhC-trnV*
Indel_230	2	Inter-species	*ndhC-trnV*
Indel_231	3	Inter-species	*ndhC-trnV*
Indel_232	11	Inter-species	*ndhC-trnV*
Indel_233	1	Inter-species	*ndhC-trnV*
Indel_234	11	Inter-species	*ndhC-trnV*
Indel_235	3	Inter-species	*trnM-atpB*
Indel_236	22	Inter-species	*trnM-atpB*
Indel_237	4	Inter-species	*atpE-rbcL*
Indel_238	65	Inter-species	*atpE-rbcL*
Indel_239	1	Inter-species	*atpE-rbcL*
Indel_240	9	Inter-species	*atpE-rbcL*
Indel_241	4	Inter-species	*atpE-rbcL*
Indel_242	5	Inter-species	*rbcL-accD*
Indel_243	8	Inter-species	*rbcL-accD*
Indel_244	7	Inter-species	*accD*
Indel_245	104	Inter-species	*accD-ycf1*
Indel_246	1	Inter-species	*accD-ycf1*
Indel_247	2	Inter-species	*accD-ycf1*
Indel_248	27	Inter-species	*accD-ycf1*
Indel_249	2	Inter-species	*accD-ycf1*
Indel_250	8	Inter-species	*accD-ycf1*
Indel_251	5	Inter-species	*accD-ycf1*
Indel_252	21	Inter-species	*accD-ycf1*
Indel_253	10	Inter-species	*accD-ycf1*
Indel_254	29	Inter-species	*accD-ycf1*
Indel_255	5	Inter-species	*accD-ycf1*
Indel_256	1	Inter-variety	*accD-ycf1*

Indel_257	18	Inter-species	*accD-ycf1*
Indel_258	1	Inter-species	*accD-ycf1*
Indel_259	11	Inter-species	*accD-ycf1*
Indel_260	14	Inter-species	*accD-ycf1*
Indel_261	2	Inter-species	*accD-ycf1*
Indel_262	3	Inter-species	*accD-ycf1*
Indel_263	1	Inter-species	*accD-ycf1*
Indel_264	3	Inter-species	*accD-ycf1*
Indel_265	21	Inter-species	*accD-ycf1*
Indel_266	3	Inter-species	*accD-ycf1*
Indel_267	3	Inter-species	*accD-ycf1*
Indel_268	15	Inter-species	*ycf1*
Indel_269	21	Inter-species	*ycf1*
Indel_270	3	Inter-species	*ycf1*
Indel_271	12	Inter-species	*ycf1*
Indel_272	9	Inter-species	*ycf1*
Indel_273	12	Inter-species	*ycf1*
Indel_274	33	Inter-species	*ycf1*
Indel_275	3	Inter-variety	*ycf1*
Indel_276	9	Inter-species	*ycf1*
Indel_277	3	Inter-species	*ycf1*
Indel_278	9	Inter-species	*ycf1*
Indel_279	3	Inter-species	*ycf1*
Indel_280	9	Inter-species	*ycf1*
Indel_281	12	Inter-species	*ycf1*
Indel_282	1	Inter-species	*trnP-trnL*
Indel_283	3	Inter-species	*trnP-trnL*
Indel_284	3	Inter-species	*trnL-chlN*
Indel_285	1	Inter-species	*trnL-chlN*
Indel_286	7	Inter-species	*trnL-chlN*
Indel_287	9	Inter-species	*chlL-trnH*
Indel_288	9	Inter-species	*chlL-trnH*
Indel_289	188	Inter-species	*chlL-trnH*
Indel_290	4	Inter-species	*chlL-trnH*
Indel_291	4	Inter-species	*trnH-trnI*
Indel_292	13	Inter-species	*trnH-trnI*

Supplementary Table 3. Nuclear 35S indels found to discriminate *Chamaecyparis* species			
Indel ID	Indel length (bp)	Indel type	Location
Indel_1	1	Inter-species	5'ETS
Indel_2	5	Inter-species	5'ETS
Indel_3	3	Inter-species	5'ETS
Indel_4	2	Inter-species	5'ETS
Indel_5	2	Inter-species	5'ETS
Indel_6	2	Inter-species	5'ETS
Indel_7	1	Inter-species	5'ETS
Indel_8	1	Inter-species	5'ETS
Indel_9	12	Inter-species	5'ETS
Indel_10	1	Inter-species	5'ETS
Indel_11	10	Inter-species	5'ETS
Indel_12	1	Inter-species	5'ETS
Indel_13	16	Inter-species	5'ETS
Indel_14	1	Inter-species	5'ETS
Indel_15	3	Inter-species	5'ETS
Indel_16	4	Inter-species	5'ETS
Indel_17	2	Inter-species	5'ETS
Indel_18	1	Inter-species	5'ETS
Indel_19	2	Inter-species	ITS1
Indel_20	1	Inter-species	ITS1
Indel_21	1	Inter-species	ITS2
Indel_22	1	Inter-species	ITS2
Indel_23	1	Inter-species	3'ETS
Indel_24	1	Inter-species	3'ETS
Indel_25	7	Inter-species	3'ETS
Indel_26	14	Inter-species	3'ETS
Indel_27	9	Inter-species	3'ETS
Indel_28	3	Inter-species	3'ETS
Indel_29	4	Inter-species	3'ETS
Indel_30	6	Inter-species	3'ETS
Indel_31	8	Inter-species	3'ETS
Indel_32	13	Inter-species	3'ETS
Indel_33	17	Inter-species	3'ETS

Indel_34	9	Inter-species	3'ETS
Indel_35	5	Inter-species	3'ETS
Indel_36	3	Inter-species	3'ETS
Indel_37	21	Inter-species	3'ETS
Indel_38	2	Inter-species	3'ETS
Indel_39	3	Inter-species	3'ETS
Indel_40	6	Inter-species	3'ETS
Indel_41	3	Inter-species	3'ETS
Indel_42	20	Inter-species	3'ETS
Indel_43	4	Inter-species	3'ETS
Indel_44	7	Inter-species	3'ETS
Indel_45	1	Inter-species	3'ETS
